# Supplementary material for: Accelerating lithium-mediated nitrogen reduction through an integrated palladium membrane hydrogenation reactor
Source: Nat Commun. 2025 Jul 28;16:6696. doi: 10.1038/s41467-025-62088-z (PMC12304132; doi:10.1038/s41467-025-62088-z)
Supplement: Supplementary file 1 — Supplementary Information [file 41467_2025_62088_MOESM1_ESM.pdf]

Supplementary information

Accelerating lithium-mediated nitrogen reduction through an integrated palladium membrane hydrogenation reactor

Hossein Bemana[1], Hendrik Schumann[1], Morgan McKee[1], Senada Nozinovic[1], Jörg Daniels[1], Ralf Weisbarth[1], Nikolay Kornienko[1]

[1]Institute of Inorganic Chemistry, University of Bonn, Gerhard-Domagk-Strasse 1, 53121 Bonn, Germany.

Table S1 - Chemicals used in this study

Name	CAS number	Purity	Manufacturer
Palladium foil	744-05-3	99.9% (25μm thickness)	Thermo Scientific
Tetrahydrofuran	109-99-9	≥99.8% (max. 0.01% H_2O)	VWR Chemicals
Lithium tetrafluoroborate	14283-07-9	98%	Abcr
Ethanol	64-17-5	≥99.8%	Fisher Scientific
Sulfuric acid	7664-93-9	95%	VWR Chemicals
Nitrogen gas	7727-37-9	99.999%	Air Liquid
Tetrahydrofuran-d_8	1693-74-9	99.5%	Deutero
Ethanol-d_6	1516-08-1	99%	Carl Roth
Deuterium oxide	7789-20-0	99.9%	Deutero
Sulfuric acid-d_2	13813-19-9	99.5 atom % D	Merck
Dimethyl sulfoxide-d_6	2206-27-1	99.8%	Deutero
Maleic acid	110-16-7	99%	Thermo Scientific
Methanol	67-56-1	99.9%	VWR Chemicals
1-propanol	71-23-8	99%	Acros Organics
1-butanol	71-36-3	≥99.5%	Sigma Aldrich
1-hexanol	111-27-3	99%	Sigma Aldrich
1,2-propanediol	57-55-6	99%	Sigma Aldrich

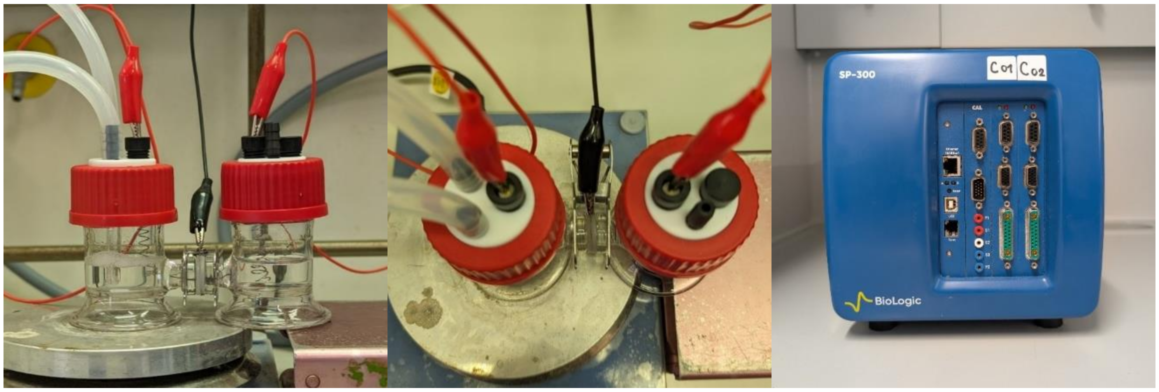

Figure S1. Digital images of the PMR-LiNRR reaction setup.

a.

b.

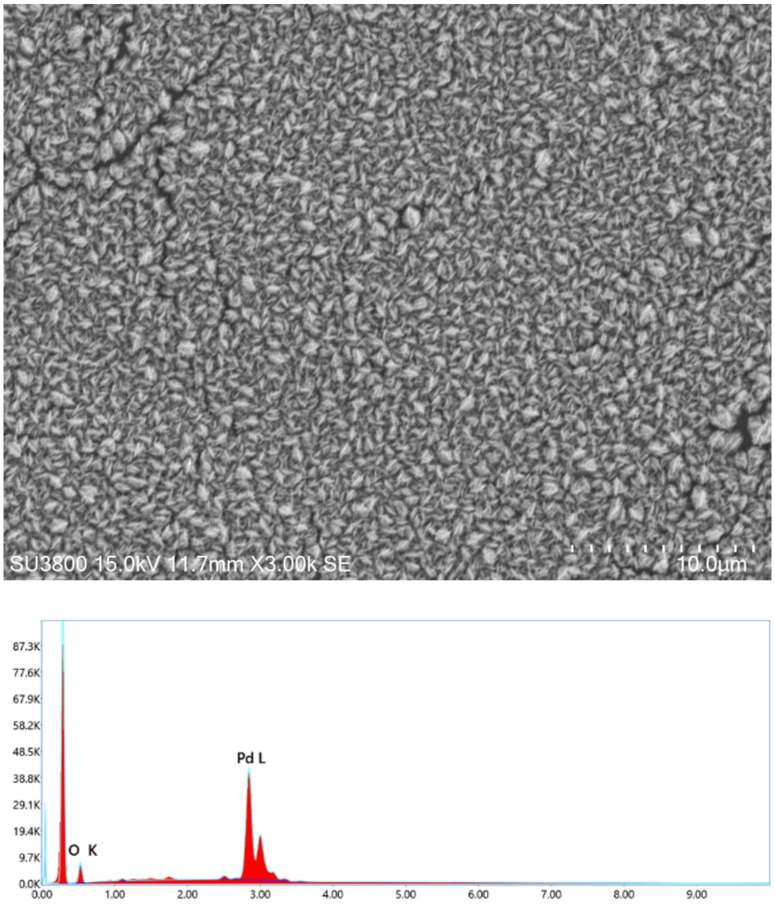

Figure S2. (a) SEM image, and (b) EDX elemental analysis of Pd foil palladized through electrochemical Pd deposition.

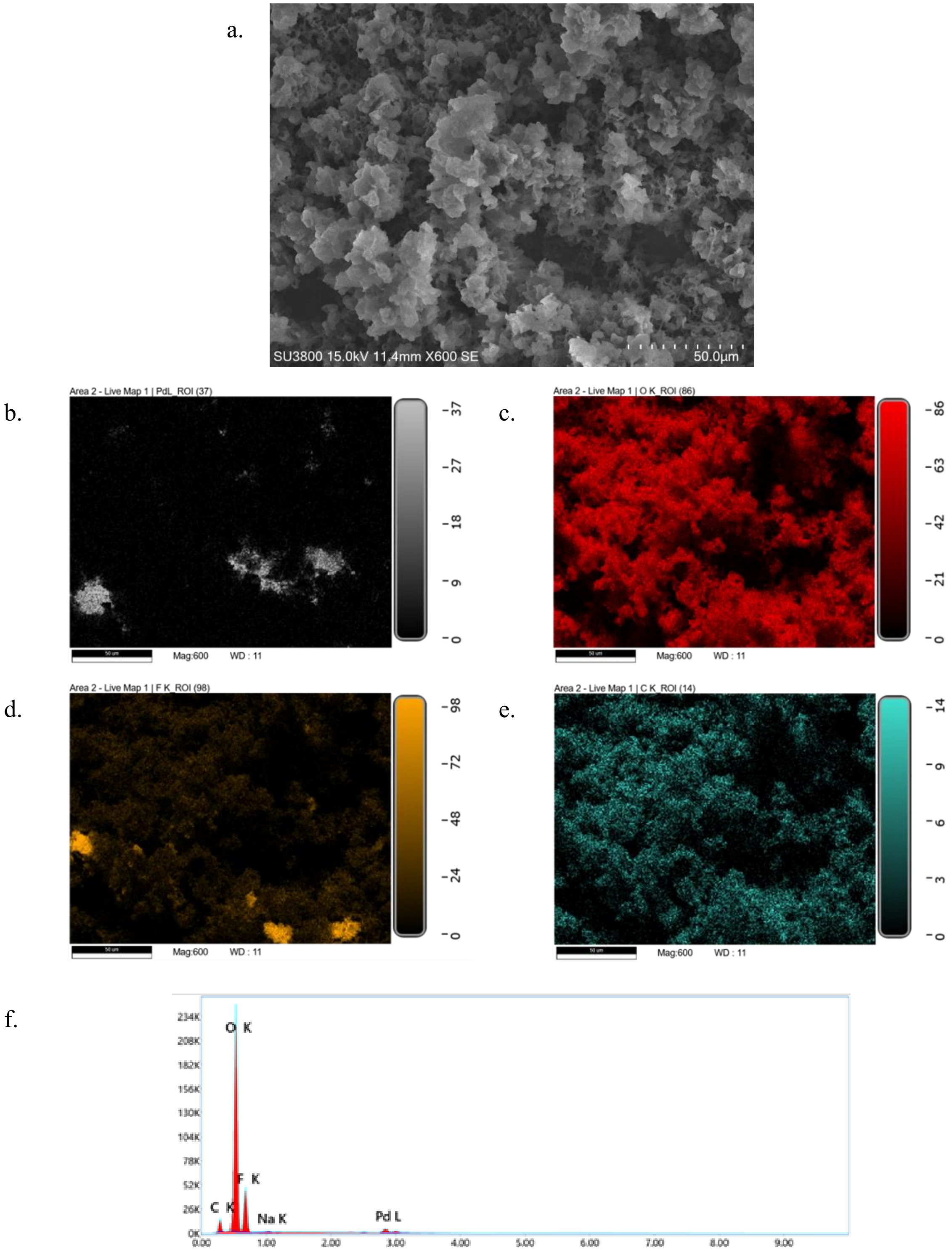

Figure S3. (a) SEM image of SEI formed on Pd foil after reaction, and EDX elemental mapping on the same region for (b) palladium, (c) oxygen, (d) fluorine, (e) carbon, (f) elemental analysis.

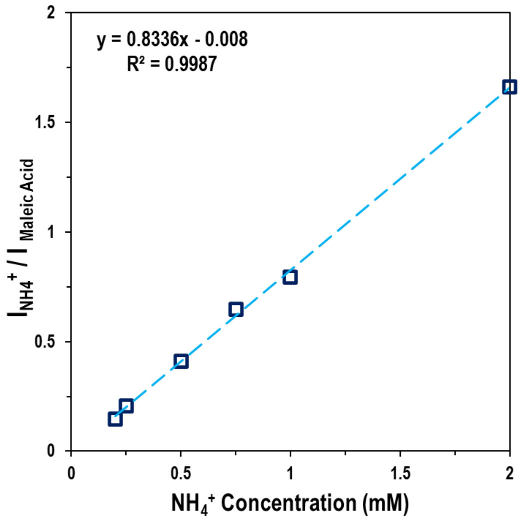

Figure S4. NMR calibration line for NH_3 quantification

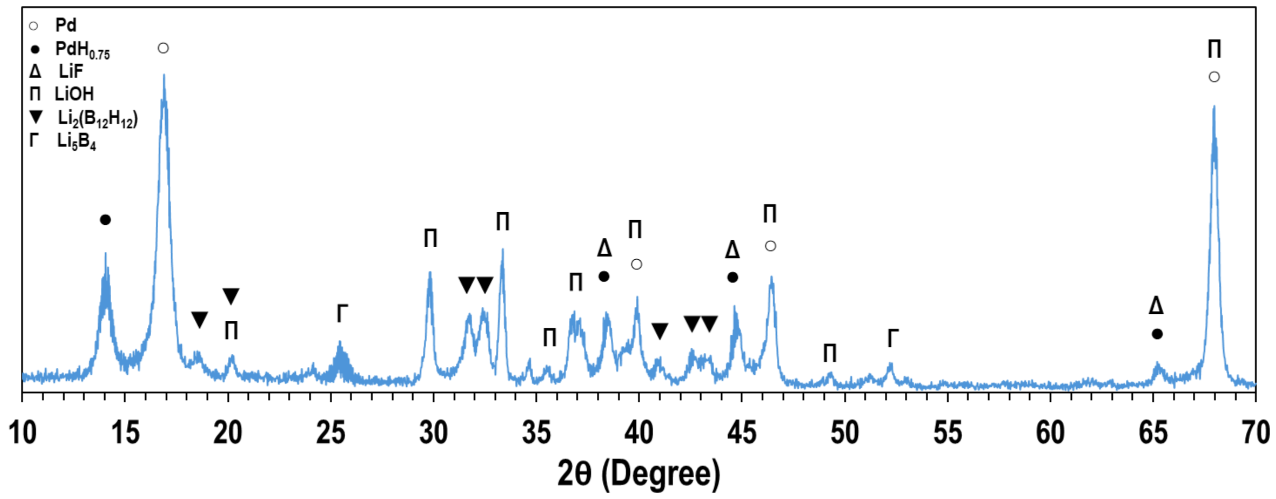

Figure S5. XRD of the air exposed SEI formed after 1 hr reaction showing Pd (○, 96-153-4922), $PdH_{0.75}$ (●, 01-082-7053), LiF (Δ 01-071-3743), LiOH (Π, 00-001-1021), $Li_2B_{12}H_{12}$ (▼, 01-077-4740), and Li_5B_4 (Γ, 00-043-0878).

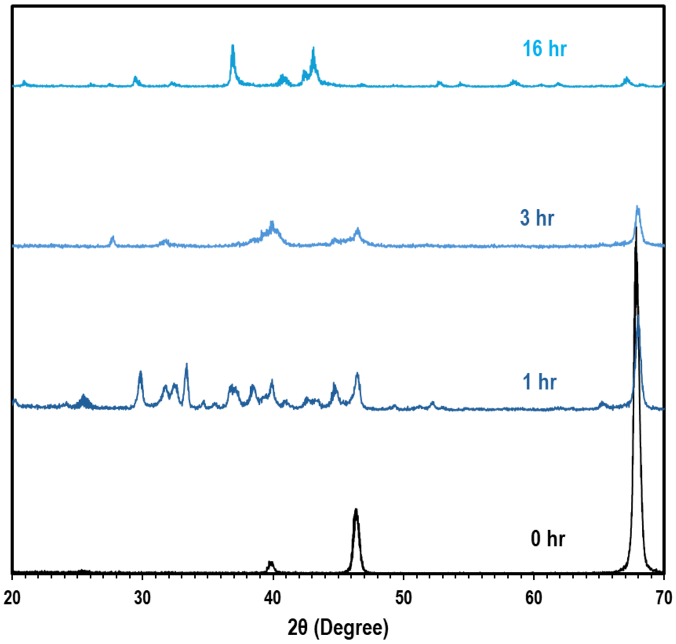

Figure S6. XRD of air exposed SEI after 0 hr (Pd foil), 1hr, 3 hr, and 16 hr reaction, reflecting the dynamic nature of SEI.

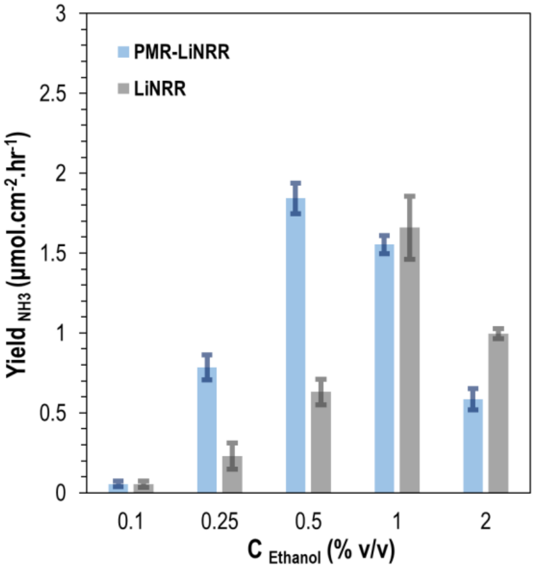

Figure S7. The effect of ethanol content on the yield of LiNRR and PMR-LiNRR. At low concentrations of ethanol, the reaction is limited by the H-donor and here we see the biggest effects of the PMR enhancement

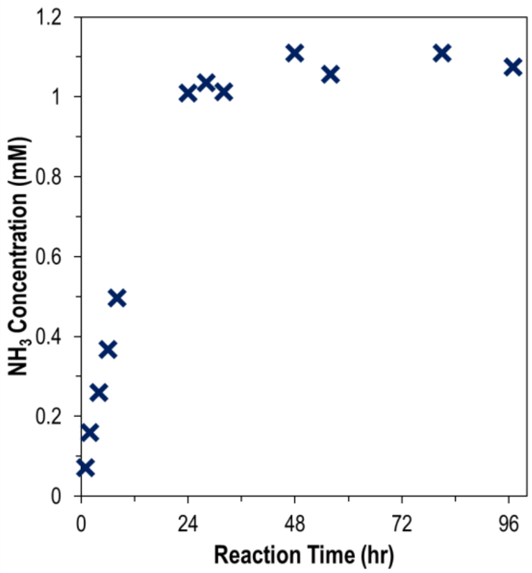

Figure S8. Variations in ammonia concentration in PMR-LiNRR during four days of operation.

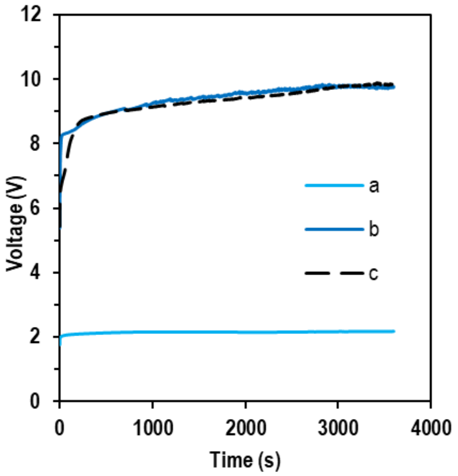

Figure S9. Voltage changes between Pd foil cathode and Pt anode while passing fixed current of 3 mA.cm^{-2} for (a) PMR side of PMR-LiNRR, (b) LiNRR side of PMR-LiNRR, and (c) LiNRR with empty PdM chamber. The composition of LiNRR chamber was 1M $LiBF_4$ + 0.5% ethanol in THF, and the PMR chamber contained 1M H_2SO_4.

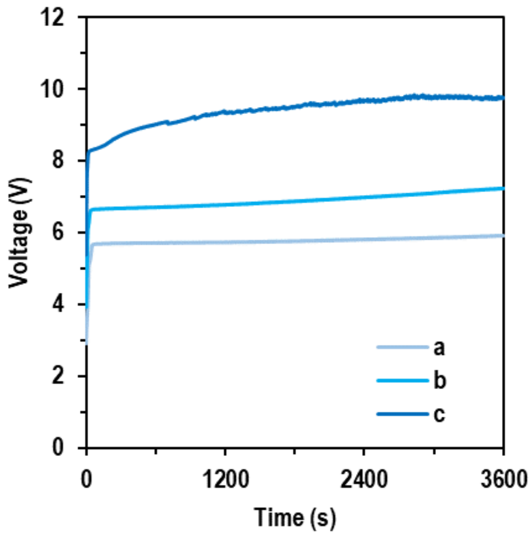

Figure S10. Voltage changes between Pd foil cathode and Pt anode of PMR-LiNRR system with fixed J_{PMR}=3 mA.cm^{-2} while J_{LiNRR} was kept at (a) 1 mA.cm^{-2}, (b) 2 mA.cm^{-2}, and (c) 3 mA.cm^{-2}. The composition of LiNRR chamber was 1M $LiBF_4$ + 0.5% ethanol in THF, and the PMR chamber contained 1M H_2SO_4.

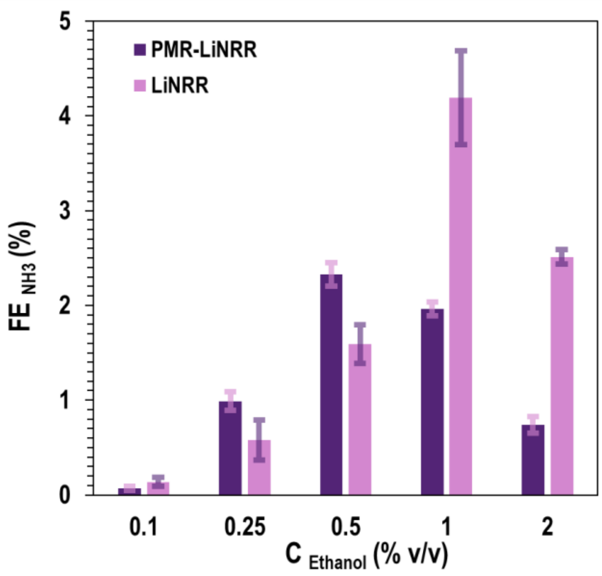

Figure S11 - The effect of ethanol content on the FE of LiNRR and PMR-LiNRR.

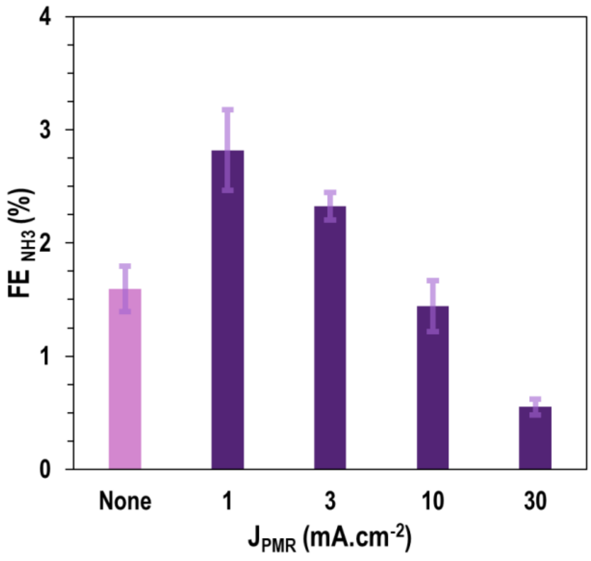

Figure S12 – The dependance of FE on the current density passing through the aqueous chamber of the PMR-LiNRR system (J_{PMR}).

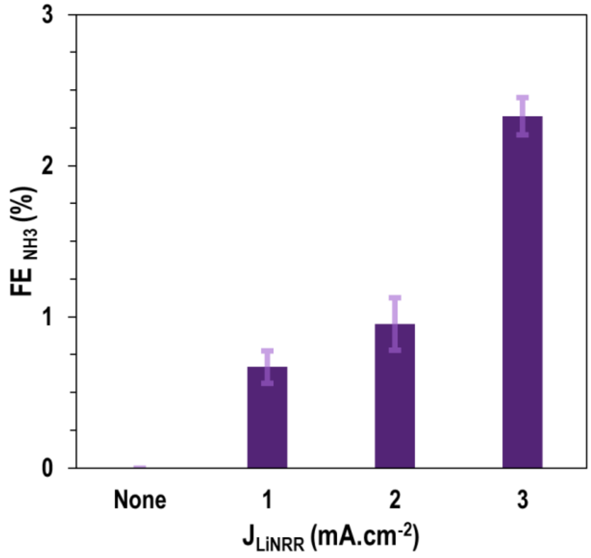

Figure S13 – The dependance of FE on the current density passing through the organic chamber of the PMR-LiNRR (J_{LiNRR}).

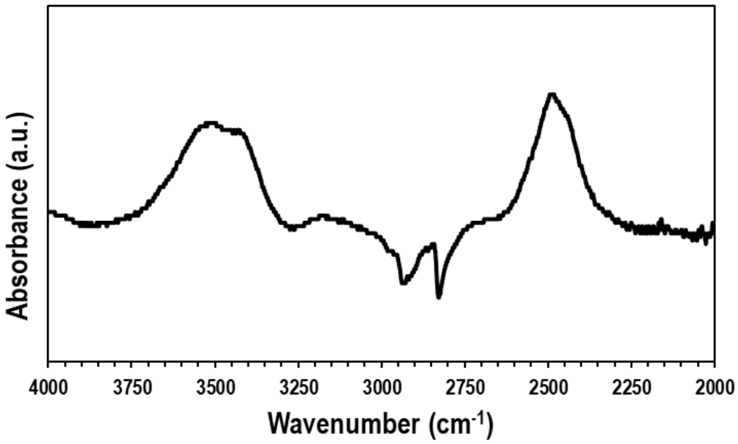

Figure S14 – FTIR spectra of SEI dissolved in methanol. The organic chamber was filled with 1M $LiBF_4$ + 0.5% ethanol in THF, and the aqueous chamber was filled with 1M D_2SO_4 in D_2O.

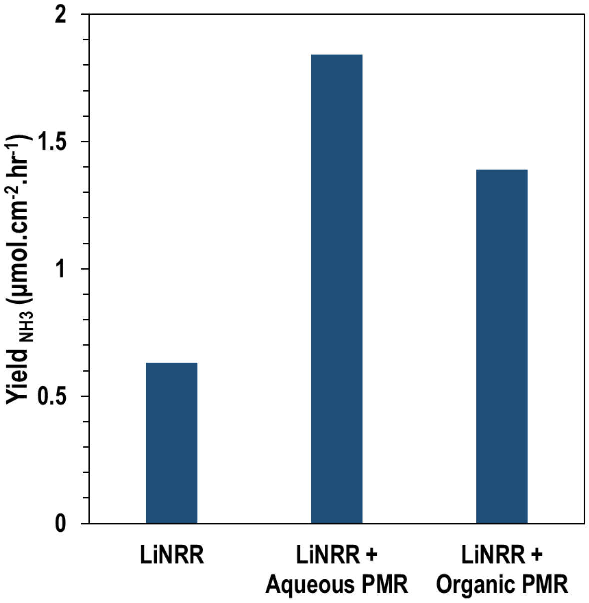

Figure S15 – Yield rate of PMR-LiNRR when PMR chamber is filled with 1M salicylic acid and 0.1 M tetrabutylammonium perchlorate dissolved in dimethoxyethane (organic PMR), compared to LiNRR (no PMR) and PMR-LiNRR filled with 1M H_2SO_4 (aqueous PMR). The current densities were J_{LiNRR} = 3 $mA.cm^{-2}$ and J_{PMR} = 3 $mA.cm^{-2}$.
